# The reversible inhibitor SR-4835 binds Cdk12/cyclin K in a noncanonical G-loop conformation

**DOI:** 10.1016/j.jbc.2023.105501

**Published:** 2023-11-26

**Authors:** Maximilian Schmitz, Ines H. Kaltheuner, Kanchan Anand, Robert Düster, Jonas Moecking, Andrii Monastyrskyi, Derek R. Duckett, William R. Roush, Matthias Geyer

**Affiliations:** 1 Institute of Structural Biology, University of Bonn, Bonn, Germany; 2 Department of Drug Discovery, Moffitt Cancer Center, Tampa, Florida, USA; 3 Department of Chemistry, The Scripps Research Institute, Jupiter, Florida, USA

**Keywords:** Cdk12, Cdk13, cyclin K, SR-4835, abemaciclib, dinaciclib, ribociclib, trilaciclib, THZ1, THZ531, NVP-2, Cdk10, HIPK3, DYRK1A

## Abstract

Inhibition of cyclin-dependent kinases (CDKs) has evolved as an emerging anticancer strategy. In addition to the cell cycle-regulating CDKs, the transcriptional kinases Cdk12 and Cdk13 have become the focus of interest as they mediate a variety of functions, including the transition from transcription initiation to elongation and termination, precursor mRNA splicing, and intronic polyadenylation. Here, we determine the crystal structure of the small molecular inhibitor SR-4835 bound to the Cdk12/cyclin K complex at 2.68 Å resolution. The compound’s benzimidazole moiety is embedded in a unique hydrogen bond network mediated by the kinase hinge region with flanking hydroxy groups of the Y815 and D819 side chains. Whereas the SR-4835 head group targets the adenine-binding pocket, the kinase’s glycine-rich loop is shifted down toward the activation loop. Additionally, the αC-helix adopts an inward conformation, and the phosphorylated T-loop threonine interacts with all three canonical arginines, a hallmark of CDK activation that is altered in Cdk12 and Cdk13. Dose-response inhibition measurements with recombinant CMGC kinases show that SR-4835 is highly specific for Cdk12 and Cdk13 following a 10-fold lower potency for Cdk10. Whereas other CDK-targeting compounds exhibit tighter binding affinities and higher potencies for kinase inhibition, SR-4835 can be considered a selective transcription elongation antagonist. Our results provide the basis for a rational improvement of SR-4835 toward Cdk12 inhibition and a gain in selectivity over other transcription regulating CDKs.

Cyclin-dependent kinases (CDKs) are serine/threonine-directed protein kinases that tightly regulate critical cellular processes. Upon activation by their corresponding cyclin subunit, they maintain cell cycle checkpoints and key transcriptional events in higher eukaryotes (1). Whereas CDKs −1, −2, −4, and −6 mediate the progression of the cell cycle through its different stages, a second subfamily of CDKs with its members Cdk7, −8, −9, −12 and −13 is involved in the regulation of the gene transcription machinery (2, 3). Transcription-associated CDKs catalyze numerous phosphorylation events on the C-terminal domain (CTD) of RNA polymerase II (RNA pol II) as well as general and specific transcriptional factors (4). In mammals, the CTD of Rpb1, the largest subunit of RNA pol II, consists of 52 hepta-repeats with the consensus sequence Y_1_S_2_P_3_T_4_S_5_P_6_S_7_ (5). Transcription is coordinated by reversible posttranslational modifications, particularly phosphorylation of CTD residues, which drive the RNA pol II-mediated transcription cycle from initiation through elongation and termination (6, 7, 8, 9).

RNA pol II-dependent gene transcription starts with the Cdk8/CycC/Med12-mediated recruitment of a hypophosphorylated RNA pol II to the gene and the formation of the preinitiation complex containing mediator complex and six general transcription factors (10, 11, 12). The kinase module of the general transcription factor TFIIH comprising Cdk7/CycH/Mat1 then facilitates initiation of transcription through phosphorylation of Ser7 and Ser5 residues within CTD hepta-repeats (6, 13, 14, 15). After promoter clearance, release from promotor proximal pausing is facilitated by the action of the Cdk9/CycT1 complex that constitutes the active form of the positive transcription elongation factor b (16). Its ability to phosphorylate Ser2 and Ser5 residues within the CTD of RNA pol II releases the polymerase from transcriptional pausing and enables productive elongation (3, 17, 18). Similar to Cdk9, kinases Cdk12 and Cdk13 have been shown to affect the regulation of RNA pol II-mediated transcription elongation through their ability to phosphorylate RNA pol II CTD Ser2 residues (19, 20, 21). In addition to regulating transcription elongation, Cdk12 directly or indirectly affects several cotranscriptional and posttranscriptional processes, including RNA splicing and processing (22, 23), and DNA damage response (DDR) (20, 24, 25, 26, 27).

Dysregulation of the transcriptional machinery can lead to defective control of gene expression, thereby promoting cancer initiation and progression. As a result, cancer cells can develop a dependency on these transcriptional dysfunctions, making ‘transcriptionally addicted’ cancers vulnerable to perturbations of the gene expression program (28, 29). Hence, interference with transcription-associated CDKs by small-molecule inhibitors has recently become a focus for the development of cancer therapeutics. In particular, dysregulation of Cdk12 activity has been associated with several types of human cancers, including breast, ovarian, prostate, and gastric cancer, and has highlighted the importance of Cdk12 as a promising diagnostic biomarker and potential therapeutic target (30, 31).

Cdk12 has been shown to affect the transcriptional elongation of a subset of DDR and homologous recombination genes, thereby contributing to genomic stability (20, 24, 25, 26, 27). Consequently, perturbations in Cdk12 activity impair RNA pol II processivity and RNA processing, resulting in reduced transcription rates of long and complex DDR-associated genes, such as BRCA1/BRCA2, and increased premature transcript termination at intronic polyadenylation sites (26, 32). The first specific inhibition of Cdk12 by a small molecule compound was reported for THZ531, which was shown to covalently target a cysteine residue in the C-terminal extension segment (33). The compound was derived from THZ1, which was introduced as a Cdk7-targeting inhibitor, and later optimized for improved selectivity and potency (34, 35). The pan-CDK inhibitor CR8 was identified through database mining as a molecular glue degrader that specifically depletes CycK (36). CR8 induces the formation of a complex between Cdk12/CycK with the E3 ligase component DNA damage-binding protein 1, which was similarly reported for three other compounds (dCeMM2/3/4) that were identified in a multiomics target deconvolution campaign through chemical screening in hypo-neddylated cells (37). While the discovery of molecular glues appears to be serendipitous, the targeted degradation of Cdk12 was achieved through the rational design of a proteolytic targeting chimera (PROTAC), BSJ-4-116, which directs the kinase to the cereblon E3 ubiquitin ligase (38). The small molecule compound SR-4835 was introduced as a potent and selective reversible inhibitor of Cdk12/Cdk13, leading to deficiencies in DNA damage repair that in combination with DNA-damaging agents and poly-ADP ribose polymerase (PARP) inhibitors result in apoptosis of triple negative breast cancer cells (39).

We here analyzed the binding specificity and efficacy of SR-4835 against various CMGC kinases by biochemical and structural means. Using *in vitro* kinase assays we confirm SR-4835’s ability to selectively target Cdk12/Cdk13 over other transcriptional CDKs or the DYRK and HIP-kinase families. We determined the crystal structure of the human Cdk12/CycK heterodimer in complex with small molecule inhibitor SR-4835 at 2.68 Å resolution, indicating a specific interaction of the compound with the kinase N-lobe. We also investigated the inhibitory activity of SR-4835 against Cdk12 compared to several other compounds and showed that dinaciclib and NVP-2 were more potent inhibitors of Cdk12 kinase activity in recombinant *in vitro* kinase assays, while being less selective. Together, SR-4835 seems to be a promising inhibitor for selectively targeting Cdk12 and shows promise for future cancer therapy applications.

## Results

### SR-4835 is a highly selective Cdk12/Cdk13 inhibitor

**Figure 1:**
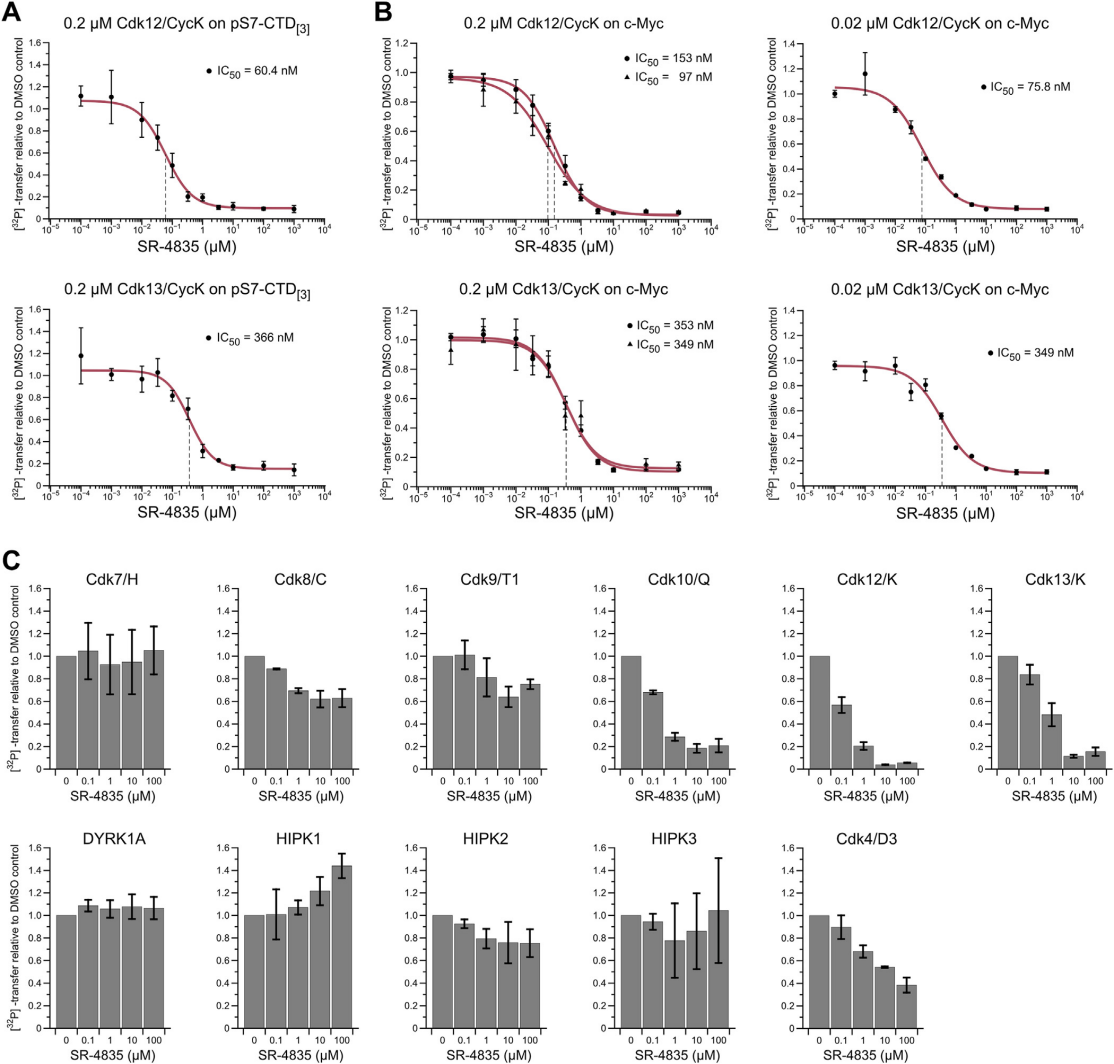
**Analysis of SR-4835 binding specificity against various CMGC kinases.***A*, dose-response curves for the inhibition of Cdk12/CycK and Cdk13/CycK by SR-4835. Radioactive kinase activity assays were performed for a concentration series of SR-4835 in presence of 0.2 μM indicated kinase and 0.2 mM [^32^P]-γ-ATP. Assays were started by addition of 100 μM pS7-CTD_[3]_ substrate and incubated for 60 min. Measurements were performed in triplicates (n = 3) and are depicted as mean ± SD. IC_50_ values were determined by sigmoidal fits and are indicated by *dashed lines*. *B*, dose-response measurements from (*A*) were repeated at 0.2 μM and 0.02 μM indicated kinase concentration on 50 μM His-c-Myc substrate for 20 min. Data are illustrated as mean ± SD from triplicates (n = 3) and IC_50_ values were determined by sigmoidal curve fitting and are indicated by *dashed lines*. *C*, SR-4835 was tested at 0, 0.1, 1, 10, and 100 μM concentration against various CMGC kinases. *In vitro* kinase assays were performed as in (*B*) at 0.2 μM indicated kinase concentration and measurements are displayed as mean ± SD from triplicates (n = 3). CDK, cyclin-dependent kinases; CTD, C-terminal domain; Cyc, cyclin.

The small molecule SR-4835, an analog of N9 heteroaromatic purines, was developed and introduced as a highly selective inhibitor targeting Cdk12 and Cdk13 with limited off-target effects (39). We tested SR-4835’s ability to inhibit Cdk12/Cdk13 *in vitro* in dose-response measurements with radioactive kinase assays using recombinant proteins. To a concentration of 0.2 μM kinase and 0.2 mM ATP, a compound concentration ranging from 0.1 nM to 1 mM was applied. Kinase reactions were started by the addition of 100 μM CTD peptide containing three hepta-repeats continuously prephosphorylated at position S7 (pS7-CTD_[3]_) and terminated after 60 min incubation time. *In vitro* IC_50_-values were determined by applying a sigmoidal fit to dose-response measurements (Fig. 1*A*). Half maximal inhibitory concentration (IC_50_) values for Cdk12/13 were in the moderate nanomolar range at 60 nM for Cdk12/CycK and 366 nM for Cdk13/CycK. Additional dose-response measurements on a surrogate c-Myc substrate at two different kinase concentrations revealed IC_50_ values within the same range as determined for the canonical CTD substrate (Fig. 1*B*). Increasing ATP concentrations to physiological levels (1 and 2 mM) confirmed SR-4835’s ATP competitive, reversible mode of action on Cdk12/CycK and Cdk13/CycK kinases (Fig. S1*A*). To account for potential tight binding affinities of SR-4835 to the kinases, we applied the Morrison quadratic equation fit to dose-response measurements at 0.2 μM and 0.02 μM Cdk12/CycK and Cdk13/CycK on c-Myc substrate (40). The Michaelis–Menten constants (*K*_M_) for ATP were determined as 17.5 μM for Cdk12/CycK and 12.6 μM for Cdk13/CycK, respectively. With these parameters and inhibitor constants (*K*_i_) calculated after Morrison’s quadratic equation, IC_50_ values in the range of 84.3 to 156 nM for Cdk12/CycK and 366 to 400 nM for Cdk13/CycK were obtained (Fig. S1*B*). These values are in accordance with the values derived from sigmoidal curve fitting.

To confirm SR-4835’s potential to selectively inhibit Cdk12 and Cdk13 of the highly conserved CDK family, we tested its inhibition potency toward various CMGC kinases. Here, SR-4835 at concentrations of 0, 0.1, 1, 10, and 100 μM was incubated for 5 min with 0.2 μM of transcription regulating CDKs Cdk7/CycH, Cdk8/CycC, Cdk9/CycT1, Cdk10/CycQ, Cdk12/CycK, and Cdk13/CycK or kinases DYRK1A, homeodomain-interacting protein kinase (HIPK) -1, -2, -3, and Cdk4/CycD3 in presence of 0.2 mM ATP and with subsequent addition of substrate (Fig. 1*C*). Whereas DYRK and HIPK protein family members were not inhibited upon addition of SR-4835, a minimal decrease in kinase activity could be observed for cell cycle-associated Cdk4/CycD3. SR-4835 displayed no inhibitory activity toward kinases Cdk7, Cdk8, and Cdk9, while a moderate SR-4835 concentration dependent decrease in Cdk10/CycQ activity was seen. To further investigate the inhibition of Cdk10/CycQ activity, a concentration response measurement was conducted using a concentration range from 1 nM to 1 mM and a sigmoidal fit was utilized to determine the *in vitro* IC_50_-value (Fig. S1*C*). A low inhibitory effect of SR-4835 toward Cdk10/CycQ with an IC_50_-value of 1070 nM was determined, a value more than 10-fold higher compared to the inhibition of Cdk12.

### Crystal structure of Cdk12/CycK bound to SR-4835

**Figure 2:**
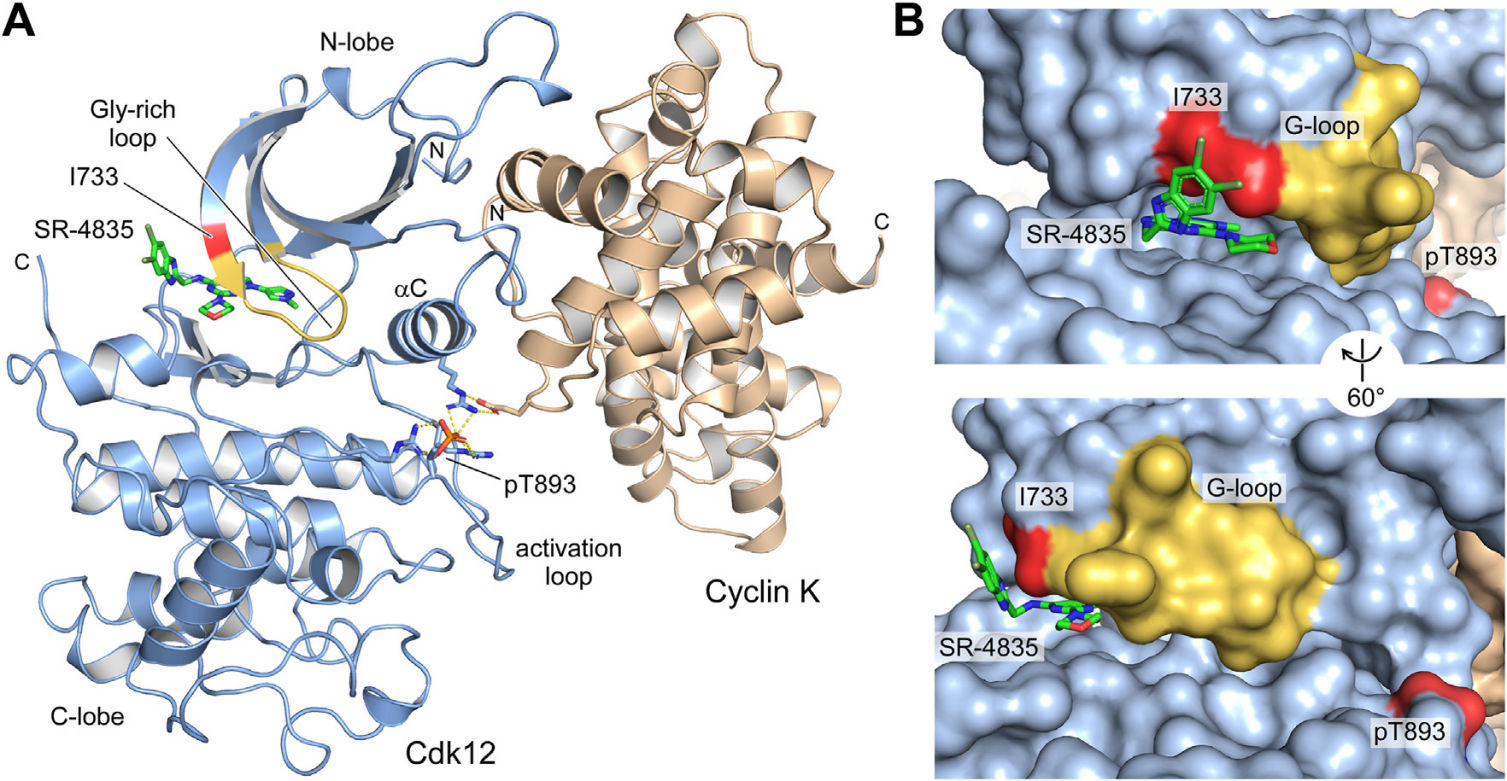
**Crystal structure of Cdk12•SR-4835/CycK complex.***A*, crystal structure of Cdk12 (*light blue*) and cyclin K (*beige*) with indicated bound small molecule inhibitor SR-4835 at 2.68 Å resolution. Key features such as the αC helix, the activation loop, and the phosphorylated threonine are highlighted. *B*, surface representation of Cdk12/CycK showing small molecule inhibitor SR-4835 binding in the kinase active site. Residue I733 of the β1-sheet of the kinase displays the largest buried surface area (BSA) of all SR-4835-interacting residues and is highlighted in *red*. CDK, cyclin-dependent kinases; Cyc, cyclin.

To understand the molecular mechanism underlying the selectively of SR-4835 to inhibit only Cdk12 and Cdk13 within the highly conserved CDK family, and to analyze the interactions between SR-4835 and Cdk12 in detail, we crystallized the complex of Cdk12/CycK bound to SR-4835 and determined the structure at 2.68 Å resolution (Table S1). The kinase fold consists of an N-terminal lobe (716–816), a C-terminal lobe (817–1020), and the C-terminal extension of Cdk12 that is continuously visible in the electron density map up to position 1035 (Fig. 2*A*). The N-lobe interacts with the first cyclin box repeat of CycK. However, there are significant differences in the orientation of the CDK/cyclin subunits relative to each other, which prevented a molecular replacement search with the prototypical Cdk12•ADP•AlF_3_/CycK crystal structure (Protein Data Bank accession code 4NST) (41). These differences relate to a twist in the kinase N-lobe relative to the C-lobe that involves also the cyclin subunit and is best seen in the lowering of the glycine-rich loop (G-loop, residues G734-Q740) toward the activation loop, leading to a closure of the kinase active site (Fig. 2*B*). Despite this difference, Cdk12 adopts an active kinase conformation as judged from the orientation of the αC-helix, the ^877^DFG-motif, and the activation loop with a phosphorylated threonine (pT893) in the T-loop. The molecular inhibitor SR-4835 and the conserved threonine residue within the activation loop in its phosphorylated form (pT893) can be clearly visualized within the electron density map (Fig. S2, *A* and *B*).

### SR-4835 is embedded in a specific hydrogen-bond network

**Figure 3:**
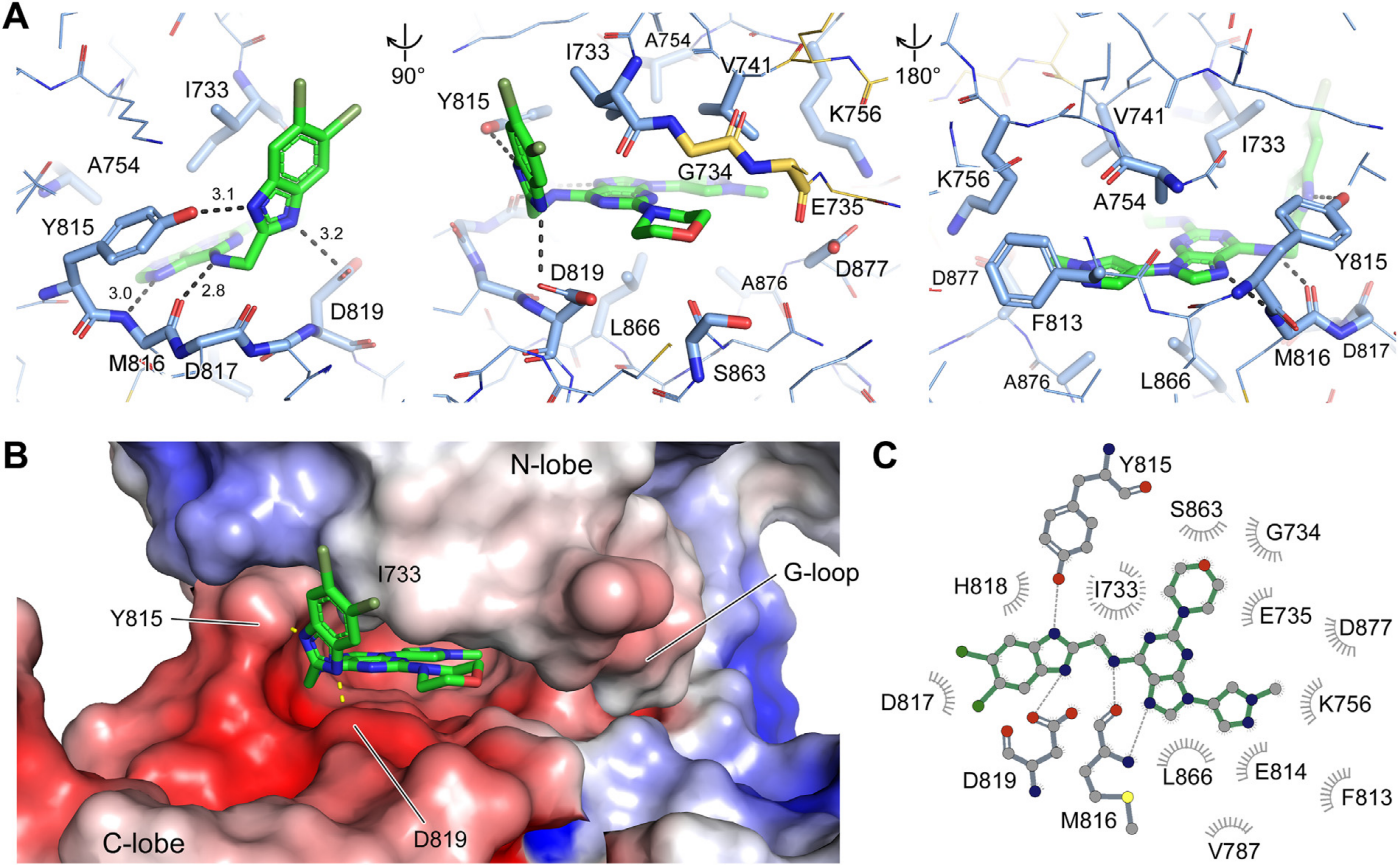
**Molecular interactions of SR-4835 with Cdk12.***A*, three close-ups of the interaction mode of SR-4835 to Cdk12. All residues interacting with the compound SR-4835 are highlighted and hydrogen bonds are represented as *black dotted lines* with the distances indicated in Å. *B*, display of the electrostatic surface potential of Cdk12 in complex with SR-4835 revealing a largely negatively charged binding pocket. The electrostatic surface charge is shown from −5 *k*_B_T (*red*) to +5 *k*_B_T (*blue*). *C*, LigPlot of the binding interface between SR-4835 and Cdk12 with indicated residues contributing direct hydrogen bonds (*dotted lines*) and residues involved in hydrophobic interactions (*gray circles* around residue) (59). CDK, cyclin-dependent kinases; Cyc, cyclin.

SR-4835 occupies the adenine-binding pocket of the kinase with its central purine- and pyrazole-moieties, while the dichloro-benzimidazole moiety of the compound sticks out of the ATP-binding pocket and winds around the first β-strand (β1) of the kinase N-lobe. As an ATP-competitive drug, SR-4835 interacts heavily with its central purine- and benzimidazole groups with the hinge region of Cdk12 (Fig. 3*A*). Hydrogen bonds are formed between the backbone amino and carbonyl groups of M816 in Cdk12 with two nitrogen atoms of the central purine moiety (Fig. 3*A*, left panel). The following benzimidazole unit of SR-4835 is additionally sandwiched between the side chains of Y815 and D819, flanking the central M816 in the hinge region. Here, two H-bonds from the hydroxy groups of residues Y815 and D819 form a clamp to the two nitrogen atoms on either side of the benzimidazole ring (Fig. 3*B*). From the top, the two catalytic spine residues V741 and A754 of the N-lobe interact with the central purine and benzimidazole groups, a typical feature of type I kinase inhibitors (Fig. 3*A*, right panel, and Fig. S2*C*) (42). Additionally, F813, which is known as the gatekeeper residue in the hinge region of Cdk12, forms hydrophobic interactions with the rear side pyrazole moiety of the compound, sharing a buried surface area (BSA) of 9.9 Å^2^. Residues of the β1-strand and G-loop I733 and G734 interact intensively *via* hydrophobic interactions with the benzimidazole and morpholino group of SR-4835, respectively (Fig. 3*A*, middle panel). In fact, the highest BSA is shared with the compound for I733 at 79.9 Å^2^. Lastly, residues S863 and L866 of the C-terminal lobe contribute to the binding of the drug to Cdk12 *via* hydrophobic interactions with BSAs of 27.0 and 31.8 Å^2^, respectively. The only residue in the interface of Cdk12 to SR-4835 that is different from the respective binding site in Cdk13 is V787, which is an isoleucine in Cdk13 (Fig. S3). However, its minor contribution of less than 3 Å^2^ BSA with the terminal pyrazole-moiety is not suggested to contribute to the selective binding of these kinases to the compound. An overview of the major interactions and H-bonds formed between Cdk12 and SR-4835 is shown in Figure 3*C*.

We aligned the sequences of transcription-regulating CDKs and compared the conservation of interacting residues with SR-4835 to gain insight into the specificity of binding (Fig. S3). Of the embedding ^815^YMDxD interaction motif within the hinge region, the key residue Y815 is replaced by a phenylalanine in Cdk7 and Cdk9, rendering these kinases incapable of forming a hydrogen bond *via* the side chain hydroxy group with the benzimidazole moiety of the compound. D819 instead is conserved in all transcriptional CDKs except Cdk8. In Cdk8, Y815 is replaced by an aliphatic, hydrophobic isoleucine, likewise incapable of forming H-bonds with the inhibitor. Only Cdk10 retains this tyrosine while the adjacent F813 is changed to a methionine, which may explain its 10-fold lower affinity for the inhibitor. The key-interacting residue I733 in Cdk12/13 is conserved in Cdk10 and Cdk9, altered to valine in Cdk8, and varied to leucine in Cdk7. Overall, it seems that the interplay of interacting residues found in Cdk12/Cdk13 contribute to the specificity of the SR-4835 compound, and that this unique combination of interactions cannot be found within the other members of transcription-associated CDKs.

### Binding to SR-4835 induces a shift in the Cdk12 G-loop

**Figure 4:**
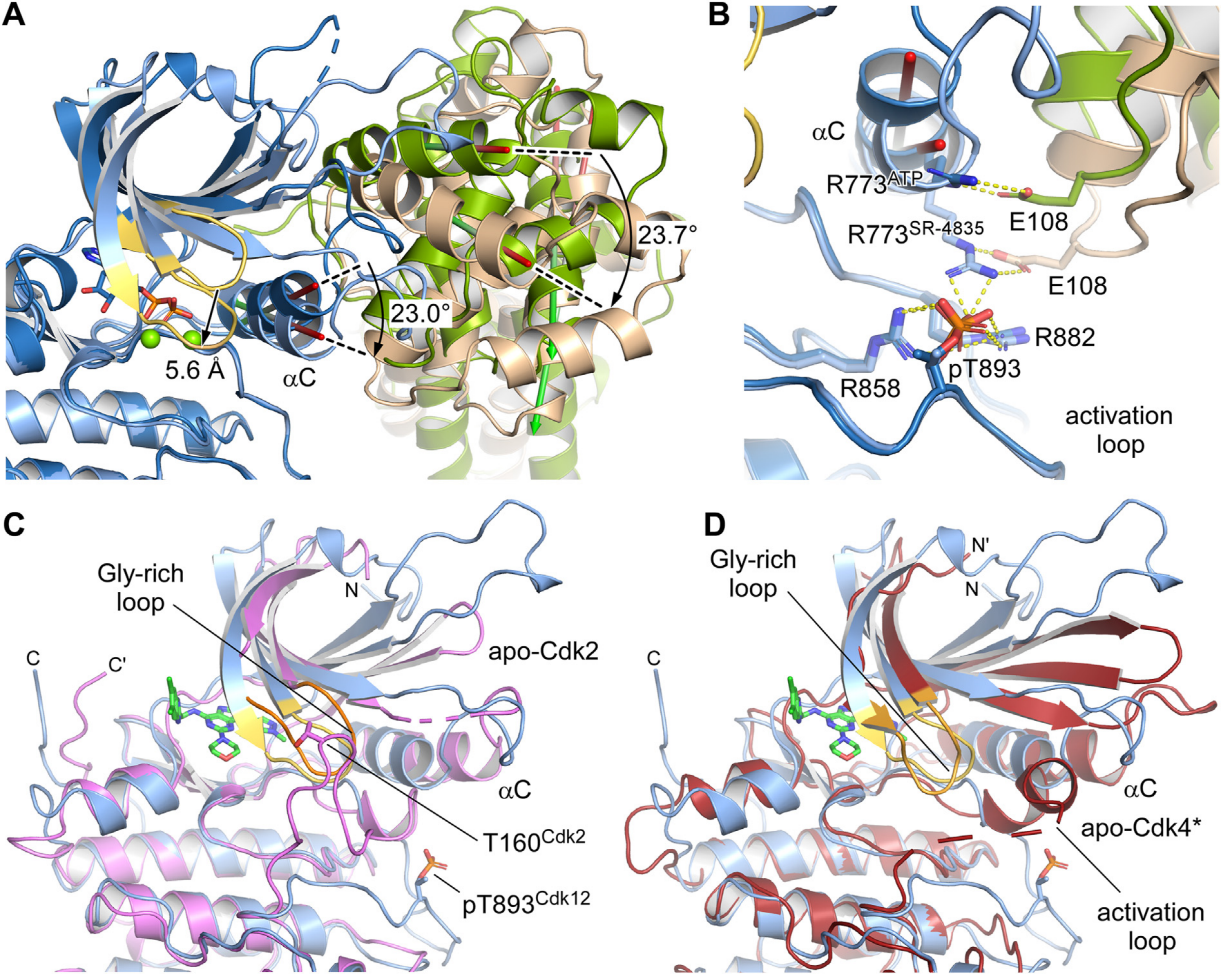
**Conformational rearrangements in Cdk12/CycK mediated by SR-4835.***A*, superimposition of the structure of Cdk12•SR-4835/CycK (8p81) to the previous structure of Cdk12•ADP•AlF_3_/CycK (4nst) on the C-lobe of the kinases reveals a twist of the cyclin subunit relative to the kinase into a more closed conformation. While the G-loop is shifted downward by 5.6 Å, the αC helix is turned in by 23°, passing through this conformational change to CycK, which is twisted by 23.7° in the SR-4835 bound conformation (*wheat*) compared to the ATP bound conformation (*green*) as displayed for the cyclin’s H5 helix. *B*, in the Cdk12•SR-4835 structure, pT893 mediates a salt-bridge network with all three canonical arginines, while in the Cdk12•ADP structure, the R773 of the PITAIRE motif solely interacts with E108 of CycK, allowing the more open conformation of the kinase-cyclin subunit assembly. *C*, overlay of the Cdk12•SR-4835 structure with the crystal structure of apo-Cdk2 (1hcl, *violet*) (44). This kinase structure in a non-T-loop phosphorylated form shows a similar conformation of the G-loop. *D*, the structure of T-loop phosphorylated Cdk4 in a nucleotide free form (2w9f) (45) exhibits an intermediate state, with the G-loop in a deepened conformation. CDK, cyclin-dependent kinases; Cyc, cyclin.

SR-4835 does not bind deep into the ATP-binding pocket of Cdk12 but rather sticks out and winds around the N-lobe of the kinase, rendering interactions between the compound and β1-strand with the G-loop important for selectivity and specificity. Molecular replacement to solve the crystal structure proved to only be successful when we separated the kinase N- and C-lobe and searched for CycK as an independent entity. The αC-helix of Cdk12 is indeed turned in by 23° relative to the Cdk12•ADP•AlF_3_/CycK structure (Fig. 4*A*). This rotational twist is accompanied by a downward shift of the G-loop of more than 5 Å, thus occupying the site that would be captured by the terminal phosphate group of ATP in an active kinase. Similar to Cdk2, the αC-helical turn allows formation of salt bridge interactions between the phosphorylated T-loop residues pT893 and arginines R773, R858, and R882 (Fig. 4*B*). This interaction network mediating kinase activation is considered a hallmark of cell cycle CDKs but was shown to be absent in transcriptional kinases Cdk12 and Cdk13 (41, 43). As a result, cyclin K moves inward toward the active center of the kinase by 23° maintaining the salt bridge of E108^CycK^ to R773^Cdk12^ (Fig. 4*A*).

The downward shift of the G-loop in the Cdk12•SR-4835 structure is rarely seen in kinases and mostly associated with a lack of T-loop phosphorylation and an inactive state. An overlay with the crystal structure of Cdk2 in the apo state and in absence of the T-loop phosphorylation (44) reveals a similar conformation as for the SR-4835-bound structure of Cdk12 determined here (Fig. 4*C*). The deep interaction of the compound with the adenine-binding pocket is reminiscent of type 1.5 kinase inhibitors, as is the interaction with catalytic spine residues A754 and V741 of the N-lobe. However, the αC "in" position and the fully active pT893 coordination give it a hybrid appearance, demonstrating once more the multiple states that kinase-inhibitor complexes can occupy. The apo structure of T-loop phosphorylated Cdk4, in which the characteristic arginine network is not formed, can be considered an intermediate conformation (45). Here, the G-loop is similarly shifted down toward the catalytic center (Fig. 4*D*). The structure of SR-4835 in complex with Cdk12/CycK thus provides insight into the specific mode of action of this novel dual Cdk12/Cdk13 inhibitor.

### SR-4835 displays moderate efficacy compared to other CDK targeting compounds

**Figure 5:**
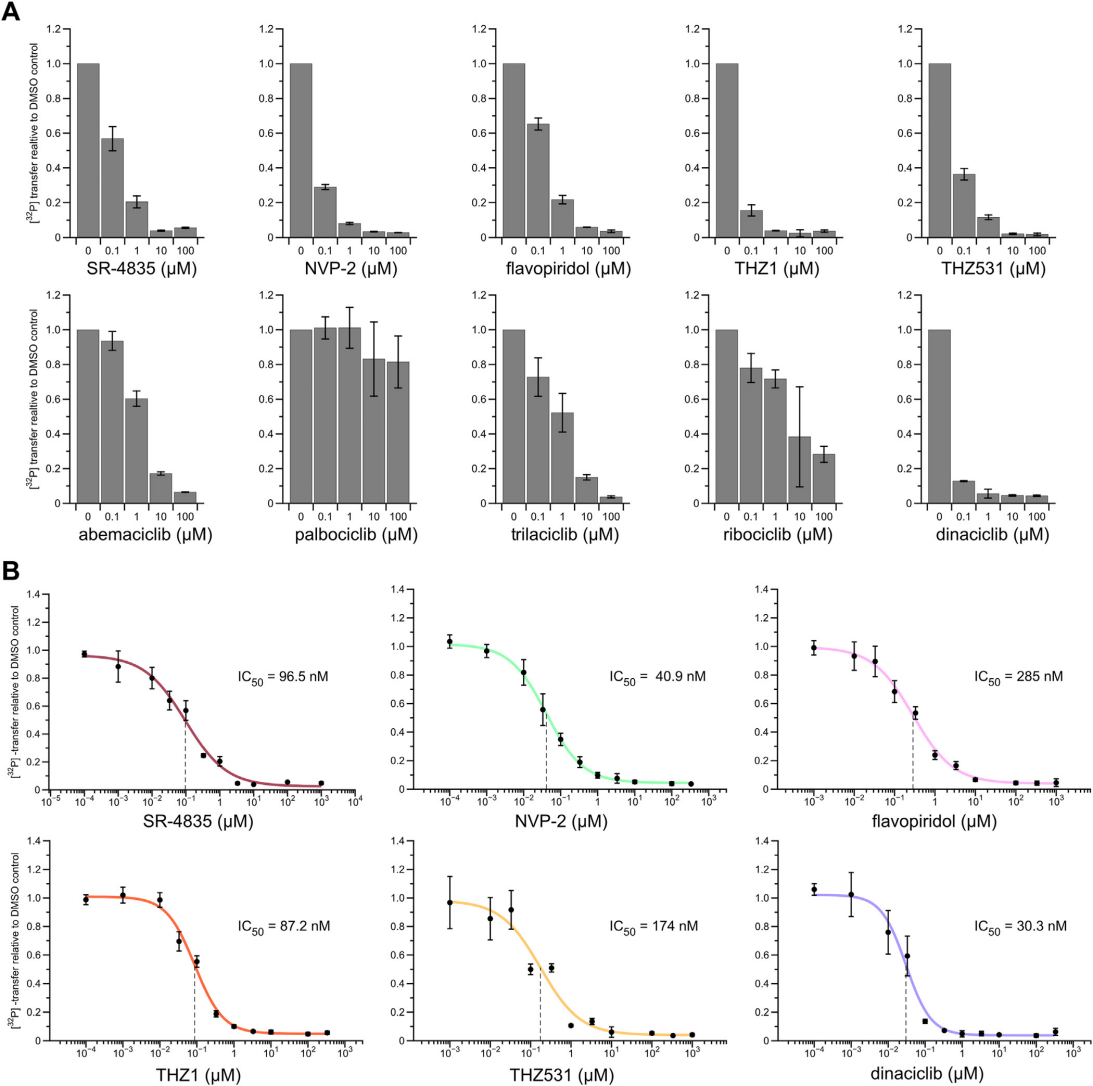
**Analysis of SR-4835 inhibition efficacy for Cdk12/CycK compared to other compounds.***A*, the efficacy of SR-4835 toward Cdk12/CycK inhibition was tested in comparison to a panel of nine small-molecule compounds. Kinase activity test measurements at 0, 0.1, 1, 10, and 100 μM respective inhibitor concentration were performed with 0.2 μM Cdk12/CycK kinase and 0.2 mM [^32^P]-γ-ATP. The substrate His-c-Myc was added at 10 μM to start the reaction, and kinase assays were incubated for 20 min. All data are displayed as mean ± SD from triplicates (n = 3). *B*, radioactive kinase activity assays were conducted as in (*A*) in dose-response measurements, testing concentration series of all small-molecules showing inhibitory effects toward Cdk12/CycK. Measurements were carried out as triplicates (n = 3) and are shown as mean ± SD. IC_50_ values were determined by sigmoidal fits and are indicated by *dashed lines*. CDK, cyclin-dependent kinases; Cyc, cyclin.

To compare the inhibition potency and efficacy of SR-4835 with other Cdk12-specific or broadly CDK-targeting compounds, we performed kinase activity measurements with selected antagonists. We choose to investigate Cdk9-targeting inhibitor NVP-2, pan-selective CDK inhibitor flavopiridol, reported covalent Cdk7-inhibitor THZ1, as well as covalent Cdk12/13-targeting inhibitor THZ531 (Fig. S4*A*). In addition, Food and Drug Administration-approved Cdk4/6 inhibitors abemaciclib, palbociclib, trilaciclib, and ribociclib were tested for their potential to inhibit Cdk12, as well as reported inhibitor dinaciclib. In a first experiment, we tested this panel of nine inhibitors plus SR-4835 to reduce Cdk12 activity *in vitro*. Cdk12 kinase (0.2 μM) in the presence of 0.2 mM ATP were supplemented with respective compounds at concentrations of 0, 0.1, 1, 10, and 100 μM for 5 min. Upon addition of 50 μM His-c-Myc substrate the kinase assay reaction was conducted for 20 min. Whereas CDK4/6 inhibitors palbociclib and ribociclib showed no inhibitory effect on Cdk12 and abemaciclib and trilaciclib displayed only weak efficacy, all other five compounds showed high inhibitory potential toward Cdk12 (Fig. 5*A*). Excluding palbociclib and ribociclib, concentration response measurements were performed for the remaining seven small molecule inhibitors to determine IC_50_ values by sigmoidal fitting (Figs. 5*B* and S4*B*). The highest efficacy toward Cdk12 was achieved by dinaciclib, a pan-CDK inhibitor, with an IC_50_ in the low nanomolar range (IC_50_ 30.3 nM). NVP-2, described as a specific and highly effective ATP-competitive Cdk9 inhibitor (46), displayed an IC_50_ of 40.9 nM toward Cdk12. THZ1 was identified to inhibit Cdk12 kinase activity with an IC_50_ value of 87.2 nM. All these above-mentioned compounds possess higher inhibitory activity for Cdk12 compared to SR-4835. THZ531, a THZ1 derived covalent inhibitor, specific for Cdk12/Cdk13 (33), and nonspecific CDK inhibitor flavopiridol reduced Cdk12 activity with moderate potency (IC_50_’s 174 nM and 285 nM, respectively). Food and Drug Administration approved Cdk4/Cdk6 inhibitor abemaciclib and trilaciclib showed only low potential to inhibit Ckd12 kinase activity, displaying IC_50_ values of 1300 and 1340 nM, respectively (Fig. S4*B*). Overall, with an IC_50_ of 97 nM, SR-4835 displays a moderate inhibiting potency toward Cdk12 compared to various other compounds that target members of the CMGC kinase group. However, based on our comparative analyses with other CDKs, SR-4835 seems to target Cdk12/13 with higher selectivity than any other reversible Cdk12-targeting inhibitor.

### SR-4835 recognizes Cdk12/Cdk13 kinase states differently

**Figure 6:**
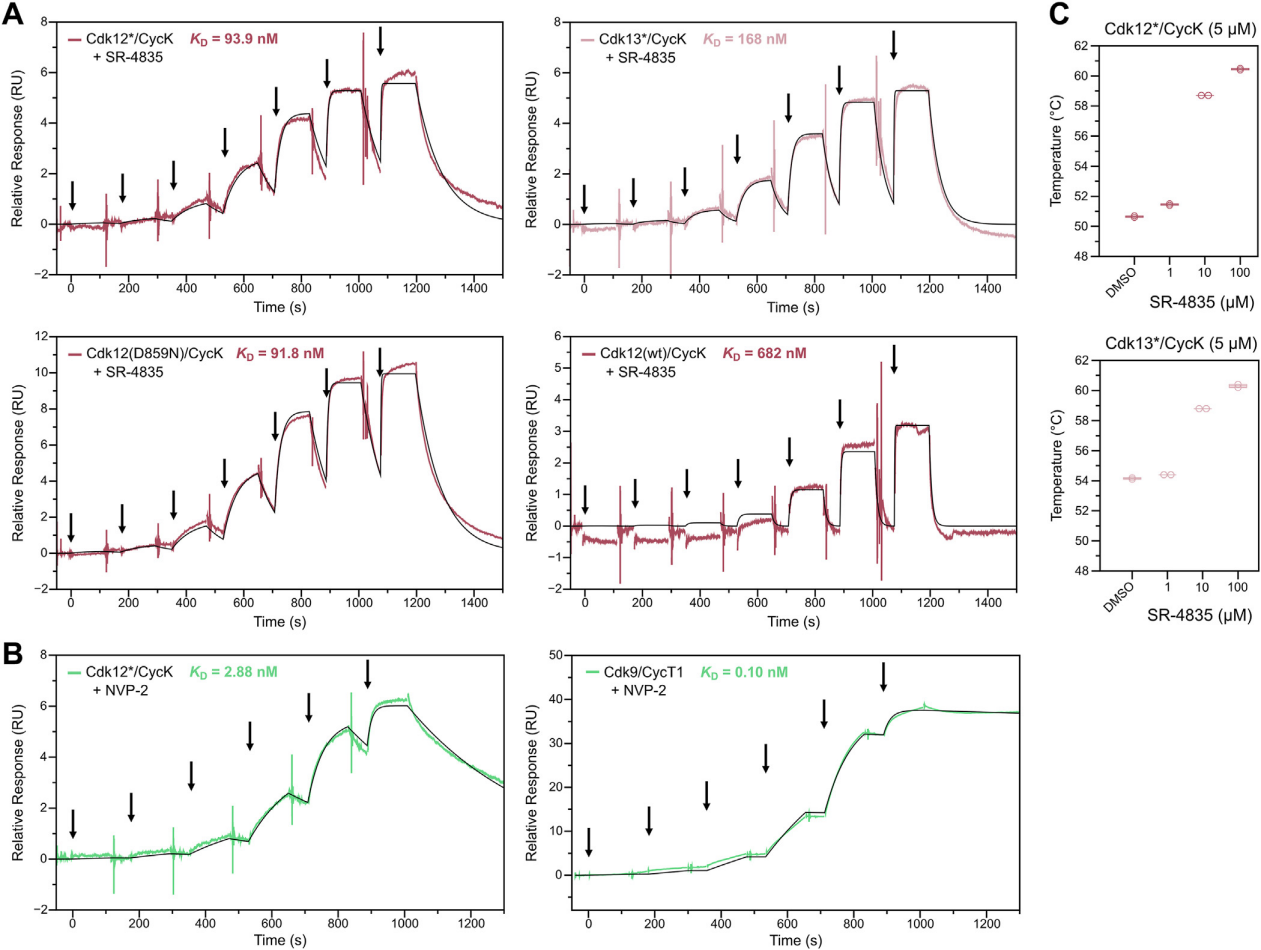
**Binding kinetics and affinity measurements of SR-4835 toward Cdk12/CycK and Cdk13/CycK.***A*, surface plasmon resonance (SPR) measurements were performed by immobilizing WT and mutant Cdk12/CycK and Cdk13/CycK to the chips surface by amine coupling. Single-cycle kinetics were measured with injections of increasing concentrations of SR-4835 (1.25, 5, 20, 80, 320, 1280, and 5120 nM) with a contact time of 120 s and a subsequent dissociation time of 300 s. Applying a general kinetics model of 1:1 binding, dissociation constants were determined. *Arrows* indicate injection of increasing SR-4835 amounts. *B*, SPR measurements were performed as in (*A*) with immobilized Cdk12/CycK or Cdk9/CycT1 and increasing concentrations of NVP-2 (0.078, 0.3125, 1.25, 5, 20, and 80 nM). A 1:1 binding model was applied for *K*_D_ determination. *Arrows* indicate injection of increasing NVP-2 amounts. *C*, nanoDSF measurements were performed to evaluate the thermal protein stability of Cdk12/CycK and Cdk13/CycK in the absence and presence of SR-4835. To a concentration of 5 μM protein kinase either DMSO or 1, 10, or 100 μM SR-4835 were added and incubated for 10 min before measurements. Box plots display the mean of two independent measurements. CDK, cyclin-dependent kinases; Cyc, cyclin; DMSO, dimethyl sulfoxide; nanoDSF, nano differential scanning fluorimetry; SPR, surface plasmon resonance.

To explore the binding kinetics and capabilities of SR-4835 toward distinct kinase states of Cdk12/CycK and Cdk13/CycK, we conducted surface plasmon resonance (SPR) experiments and thermal stability measurements. Three different kinase states were compared, including a fully T-loop phosphorylated active kinase, a kinase dead variant, and a largely T-loop unphosphorylated inactive kinase, possibly mimicking different conformations of the αC helix and the G-loop. The protein kinases were immobilized on a CM5 chip using the amine-coupling method. Single-cycle kinetic measurements were conducted with seven different concentrations of SR-4835, progressively increasing in concentration. The fully active and T-loop phosphorylated kinase preparations of Cdk12∗/CycK and Cdk13∗/CycK were obtained through coexpression with Cdk-activating kinase 1 (CAK1) from *Saccharomyces cerevisiae*. The dissociation constants (*K*_D_’s) of SR-4835 for binding to Cdk12∗/CycK of 94 nM and binding to Cdk13∗/CycK of 168 nM agree very well with the IC_50_ values of their inhibitory potential (Fig. 6*A*). A kinase dead mutant preparation of Cdk12/CycK (Cdk12 D859N/CycK), which was phosphorylated in the T-loop (Fig. S5*B*) but lacks kinase activity (Fig. S5*A*), exhibited a similar *K*_D_ of 92 nM when compared to the fully active Cdk12∗/CycK WT kinase. In contrast, when examining a Cdk12/CycK preparation that was not coexpressed with CAK1, resulting in reduced T-loop phosphorylation (Fig. S5*B*) and activity (Fig. S5*A*), an approximately 7-fold lower affinity (*K*_D_ = 682 nM) was observed. The data obtained indicate that the reduction of T-loop phosphorylation and the resulting conformational changes within the kinase fold led to a decreased binding affinity between SR-4835 and Cdk12/CycK. However, it is noteworthy that the binding of SR-4835 toward Cdk12/CycK is not completely abolished. For comparison, we measured the affinity of NVP-2 for active kinases Cdk12∗/CycK and its proposed target Cdk9/CycT1 by SPR. Here, a *K*_D_ of 2.9 nM was determined for the binding to Cdk12 but an even 20-fold lower *K*_D_ to its proposed target Cdk9 (Fig. 6*B*). These data are in agreement with the IC_50_ values determined above, confirming the high potency of the compound toward the transcription elongation kinases.

In order to assess the changes in thermal protein stability of Cdk12∗/CycK and Cdk13∗/CycK in response to increasing concentrations of SR-4835, we performed nano differential scanning fluorimetry (nanoDSF) experiments (Fig. 6*C*). Three different concentrations of SR-4835 (1, 10, and 100 μM) were added to a solution containing 5 μM of the respective protein and related to dimethyl sulfoxide (DMSO) control measurements. Upon supplementation of SR-4835, Cdk12∗/CycK exhibited notable increases in melting temperatures, resulting in thermal shifts of +8.1 °C (at 10 μM) and +9.8 °C (at 100 μM), respectively. Similarly, the addition of SR-4835 resulted in the stabilization of Cdk13∗/CycK kinase, albeit with reduced thermal shifts in melting temperatures compared to Cdk12∗/CycK. At 10 μM concentration, the thermal shift observed was +4.7 °C, while at 100 μM the thermal stability was increased by +6.2 °C (Table S2) compared to a DMSO control. Together, the results obtained from both SPR and nanoDSF measurements align well with the IC_50_ values determined for Cdk12∗/CycK and Cdk13∗/CycK kinases. These findings consistently demonstrate that SR-4835 exhibits higher affinities toward Cdk12∗/CycK in comparison to Cdk13∗/CycK.

## Discussion

CDKs have emerged as promising targets for anticancer therapies, with CDK inhibition being extensively explored and applied (29). Particularly, the roles of Cdk12 and Cdk13 in transcription regulation have generated increased attention due to their involvement in a multitude of essential processes. As a result, their potential as therapeutic targets for the treatment of various types of cancer has been identified (30, 47). Nevertheless, the development of potent inhibitors that selectively target specific members of the CDK family has proven to be challenging, as transcription-associated CDKs, much like other kinases, rely on ATP as a cofactor and possess a structurally similar active site (48). To enhance the selectivity of compounds targeting Cdk12 and Cdk13 various strategies have been employed, including development of covalent inhibitors (33, 35) and degrader compounds (36, 37, 38). In fact, SR-4835 has been described as a molecular glue in a study of colorectal cancer treatment and in the rational design of CycK degraders (49, 50). Furthermore, SR-4835 has been reported as a promising dual reversible small molecule inhibitor for Cdk12 and Cdk13 (39). However, the development of compounds that can discriminate between closely related kinases Cdk12 and Cdk13 and exclusively target one of these kinases has not been reported in the literature to date.

In this study, we determined the crystal structure of heterodimeric Cdk12/CycK complex when bound to small molecular inhibitor SR-4835. By analyzing the complex, we gain significant insights into the molecular mechanism underlying inhibition and targeting of Cdk12 and Cdk13 by SR-4835. *In vitro* dose-response inhibition measurements confirmed the findings reported by Quereda *et al.* regarding the high susceptibility of Cdk12 and Cdk13 for SR-4835 (39). Our results revealed that SR-4835 exhibited approximately 10-fold lower potency for Cdk10 compared to Cdk12 and Cdk13, while displaying no inhibitory effect on other members of the transcriptional CDK, HIPK, and DYRK families. Interestingly, at physiologic ATP concentration the potency of the inhibitor was severely diminished, contrasting the strong effect in cellular studies. We hypothesize that SR-4835’s ability to induce cyclin K degradation accounts for this apparent difference between our *in vitro* and published *in vivo* data (39). By structural analysis, a distinct hydrogen bond network mediated by the kinase hinge region was identified, effectively constricting the benzimidazole moiety of SR-4835. This unique interaction pattern involved the hydroxy groups of the side chains Y815 and D819, which further flanked the compound within the adenine binding pocket. Such a specific interplay of residues within the kinase hinge region is inimitable to Cdk12 and Cdk13 and was not observed in other members of the transcription-associated CDKs. Thus, modest structural differences within the ATP-binding pocket and hinge region of the kinases are responsible for the selectivity of SR-4835.

When analyzing the interacting residues between Cdk12 and Cdk13 with SR-4835, we observed only one change in the binding interface. Specifically, V787 in Cdk12 is replaced by isoleucine (I765) in Cdk13. While the alteration of the residue from one aliphatic hydrophobic amino acid to another may not have a substantial impact on the selectivity of the drug, our analysis revealed some notable differences between Cdk12 and Cdk13 regarding the binding of SR-4835. Specifically, we observed increased IC_50_-values, dissociation constants, and decreased thermal stabilization of SR-4835 toward Cdk13 compared to Cdk12. These findings suggest that this residue change within the interface of these kinases and the compound presents an opportunity for future improvements of SR-4835. By capitalizing on this difference, it may be possible to develop compounds that specifically target Cdk12, further distinguishing it from the highly conserved Cdk13 kinase. Importantly, the individual inhibition of either Cdk12 or Cdk13 has been observed to trigger transcriptional responses linked to cellular growth signaling pathways and/or DNA damage, while minimally effecting cell viability, RNA pol II phosphorylation levels, and RNA pol II processivity. In contrast, the simultaneous inhibition of both kinases strongly induced cell death and resulted in diminished RNA pol II CTD phosphorylation and reduced RNA pol II processivity (51). Therefore, enhancing the selectivity of compounds toward Cdk12 over Cdk13 could potentially reduce side effects and expand the therapeutic applications of these inhibitors. In conclusion, our research contributes to the understanding of the unique binding properties of SR-4835 to Cdk12. These findings could open avenues for further exploration and development of Cdk12-specific inhibitors, potentially offering new therapeutic opportunities in the field of cancer research.

## Experimental procedures

### Protein expression

Recombinant proteins were expressed either in *Sf9* insect cells (Cdk12/CycK, Cdk13/CycK, Cdk4/CycD3, Cdk7/CycH/MAT1, Cdk9, Cdk10/CycQ, HIPK1, HIPK2, HIPK3) or in *Escherichia* *coli* BL21 pLysS cells (DYRK1A, glutathione-S-transferase (GST)-CTD_[52]_, His-c-Myc, GST-retinoblastoma-associated protein 1 (Rb1), CycT1). *Sf9* cell cultures were maintained in SF-900 III *serum-free* medium (Invitrogen) at 27 °C and 80 rpm, and expression in *Sf9* insect cells was conducted using the MultiBac^Turbo^ system (52). *E. coli* (DH10) MultiBac^Turbo^ cells were transformed with respective plasmids for insertion into the baculoviral genome. Recombinant bacmid DNA was isolated and used for subsequent transfection of *Sf9* cells, followed by propagation of baculoviruses. For protein expression, *Sf9* insect cells at a density of 1.5 × 10^6^ cells/ml were infected by supplementing 2% (v/v) baculovirus V2 preparation for 72 h. Cells were collected by centrifugation at 2000 rpm (JLA8.1 rotor, Beckman Coulter), washed with PBS, snap frozen in liquid nitrogen, and stored at −80 °C for later use. For recombinant protein expression in bacteria, precultures of *E. coli* cells were grown in LB medium in the presence of appropriate antibiotics at 37 °C overnight. Precultures were used to inoculate larger volumes of LB medium to an *A*_600_ of 0.1. Cultures were grown at 37 °C until an absorbance of 0.8 was reached. Protein expression was induced by addition of IPTG to a final concentration of 0.4 mM and cells were incubated at 18 to 20 °C overnight. Bacteria were pelleted by centrifugation at 4000 rpm (JLA8.1 rotor, Beckman Coulter), washed in PBS, and either subjected to cell lysis or snap frozen in liquid nitrogen and stored at −20 °C.

### Recombinant protein kinases

The kinase domain of human Cdk12 (residues 714–1063, UniProt accession number Q9NYV4) and cyclin box domains of human cyclin K (residues 1–267, UniProt accession number O75909) were codon optimized for expression and purchased as synthetic genes from GeneArt. Cdk12 and CycK were inserted into pACEBac1 acceptor vector and pIDK donor vector, respectively. Both plasmids were similarly modified with an N-terminal GST-affinity tag followed by a tobacco etch virus (TEV) protease cleavage site. A pIDC donor vector was used to insert full-length CDK-activating kinase CAK1 (UniProt accession number P43568) from *S. cerevisiae* without an affinity tag. Cre recombination was performed for fusion of all three vectors *in vitro* and fused vectors were used for coexpression in *Sf9* insect cells. For purification of GST-Cdk12/GST-CycK protein complex, cells were resuspended in lysis buffer (50 mM Hepes pH 7.8, 300 mM NaCl, 5 mM β-mercaptoethanol (β-ME), 10% glycerol) supplemented with 1:100 (v/v) PMSF and 1:1000 (v/v) DNAse and were lysed by sonication. Disrupted cells were subjected to centrifugation at 50,000*g* for 45 min at 10 °C in a Beckman Coulter Avanti JXN-26 centrifuge. The supernatant was filtered through a syringe filter (0.45 μm) and applied to a GSTrap FF column (Cytiva) equilibrated in lysis buffer using an ÄKTA start chromatography system (GE HealthCare). After extensive washing with 10 column volumes lysis buffer and 5 column volumes wash buffer (50 mM Hepes pH 7.8, 1 M NaCl, 5 mM β-ME, and 10% glycerol), the protein complex was eluted in GST elution buffer (50 mM Hepes pH 7.8, 300 mM NaCl, 5 mM β-ME, 10 mM glutathione). Protein fractions were concentrated *via* Amicon Ultra centrifugal filters (Millipore) and subjected to TEV protease digest (1:100) at 4 °C overnight, resulting in GST-affinity tag removal. Protein samples were concentrated and loaded onto a preparative HiLoad 16/600 Superdex 200 prep grade column (Cytiva) equilibrated in size-exclusion chromatography (SEC) buffer (20 mM Hepes pH 7.8, 400 mM NaCl, 1 mM tris(2-carboxyethyl)phosphine, 5% glycerol). Fractions containing pure Cdk12/CycK protein complex monitored by SDS-PAGE were pooled, concentrated, aliquoted, snap frozen in liquid nitrogen, and stored at −80 °C. Expression constructs for Cdk12 point mutations D859N and T893A were generated by primer site-directed mutagenesis following fusion with WT CycK and CAK1 vectors, expression in *Sf9* insect cells, and purification as described for the WT protein. Additionally, a WT version of Cdk12 was fused with WT CycK without the CAK1 vector and expressed in *Sf*9 insect cells. The purification process was carried out using the same protocol as previously described (53).

The kinase domain of human Cdk13 (residues 694–1039, UniProt Q14004) was fused with plasmids of CycK and CAK1 for expression in *Sf9* insect cells and purified as described above for Cdk12. Full-length, human, WT Cdk9 (residues 1–372, UniProt P50750) was expressed in *Sf9* insect cells and purified *via* affinity chromatography and gel filtration as described (17). The cyclin boxes of human CycT1 (residues 1–272, UniProt O60563) were separately expressed in *E. coli* cells and purified by GST-affinity chromatography. Heterodimeric positive transcription elongation factor b (Cdk9/CycT1) was reconstituted by mixing both proteins in a 1:1 M ratio prior to SEC and fractionation. Full-length, WT, human Cdk4 (residues 1–303, UniProt P11802) complexed with full-length, WT, human cyclin D3 (residues 1–292, UniProt P30281) was coexpressed in *Sf9* insect cells and purified by a combination of GST-affinity chromatography, TEV-mediated affinity tag removal, and SEC as described (53).

Full-length, human, WT Cdk10 (residues 1–360, UniProt Q15131) and full-length, human, WT CycQ (residues 1–248, UniProt Q8N1B3) were coexpressed in *Sf9* insect cells, and the protein complex was purified by affinity chromatography and gel filtration as described (54). Full-length, human, WT Cdk7 (residues 1–346, UniProt P50613), full-length, human, WT CycH (residues 1–323, UniProt P51946), and a construct of human MAT1 (residues 230–309, UniProt P51948) were purchased as synthetic genes codon optimized for expression in *Spodoptera frugiperda* from GeneArt. Coexpression and affinity purification of the ternary complex was performed as described (54). The production of CMGC kinases WT, human HIPK1 (residues 154–554, UniProt Q86Z02), WT, human HIPK2 (residues 160–563, UniProt Q9H2X6), and WT, human HIPK3 (residues 159–562, UniProt Q9H422) as recombinant protein from *Sf9* insect cells was performed as described (53). The kinase domain of human DYRK1A (residues 126–485, UniProt Q13627) was expressed in *E. coli* cells and purified by sequential affinity chromatography and SEC as described (53).

### Substrate proteins

For kinase activity measurements recombinant substrate proteins c-Myc, RNA pol II CTD, and Rb1 were expressed in *E.coli* bacterial cells. The N-terminal domains of human, WT c-Myc (residues 17–167, UniProt P01106) were cloned into a pProEx-HTa backbone comprised of an N-terminal hexa-histidine affinity tag trailed by a TEV protease cleavage site and purified from inclusion bodies as described (53). The CTD including all 52 hepta-repeats of human WT RNA pol II subunit Rpb1 (residues 1587–1970, UniProt P24928) was expressed and purified with consecutive GST-affinity chromatography and gel filtration (41). Human, WT Rb1 (residues 761–928, UniProt P06400) was inserted into a pGEX-6P1 expression plasmid harboring an N-terminal affinity GST-tag followed by a TEV protease truncation site. Protein expression and purification was carried out as described above for GST-tagged proteins, except GST-tag was kept on, resulting in intact recombinant GST-Rb1 protein. A three hepta-repeat long RNA polymerase II CTD substrate that is continuously phosphorylated at position S7 (pS7-CTD_[3]_) was purchased from Biosyntan.

### Small molecular compounds

SR-4835 powder (cat# HY-130250, cas: 2387704-62-1) at a purity of 99.73% was purchased from MedChemExpress with Lot # 61794. Alternatively, SR-4835 was bought as a liquid solution dissolved at a concentration of 10 mM in 1 ml DMSO (Lot # 61794). However, repeated measurements showed irreproducible IC_50_ values, which we attributed to freeze-thaw cycles. For comparative studies, SR-4835 was purchased from Axon MedChem (Axon 3184/Batch1) or received from the Monastyrskyi laboratory at the Moffitt Cancer Center, revealing both similar inhibitory potencies. If not stated otherwise, only freshly thawed aliquots of SR-4835 from Axon MedChem were used. Compounds NVP-2, HMR-1275 (flavopiridol), THZ1, THZ531, LY2835219 (abemaciclib), PD0332991 (palbociclib), G1T28 (trilaciclib), LEE011 (ribociclib), and SCH727965 (dinaciclib) for kinase inhibition were purchased from MedChemExpress. Compounds were either bought as 10 mM stocks in DMSO or dissolved in 100% DMSO to a 10 mM stock solution.

### Protein crystallization and diffraction data collection

Cdk12/CycK protein in complex with SR-4835 was crystallized by the hanging drop vapor diffusion method at 15 °C. Purified protein was concentrated to 12 mg/ml, mixed with a 5-fold excess of the compound SR-4835 followed by 30 min incubation on ice before setting up drops for crystallization. Initial crystals of the Cdk12•SR-4835/CycK complex were obtained using a homemade PegMix screen (0.1 M Mops pH 6.0, 0.2 M MgCl_2_, 40% medium-weight PEGs). Optimal crystals were grown with a 1:1 ratio of protein and precipitant solution by using 0.1 M Mops pH 6.5, 30% PEG mix and 0.1 M NDSB. Crystals were cryoprotected with 15% ethylene glycol in the mother-liquor and flash cooled in liquid nitrogen. Data were collected from a single loop-mounted crystal held in a stream of cooled nitrogen gas at 100 K (Oxford Cryosystems). Data up to 2.75 Å resolution were collected at the P14 synchrotron beamline at Deutsches Elektronen-Synchrotron (DESY) Hamburg, Germany, equipped with an Eiger detector.

### Data processing, structure determination, and model building

Data were processed and scaled using the XDS program package (https://xds.mr.mpg.de/) (55). The phase problem was resolved by the molecular replacement method using the program PHASER (56) and the coordinates of Cdk12/CycK (4nst) (41) as a search model for the phase solution. The search model had to be truncated into the kinase N- and C-lobes in order to obtain the correct solution. The model was refined in alternating cycles with PHENIX (https://phenix-online.org/) (57). Manual rebuilding and visual comparisons were made using the graphical program COOT (https://www2.mrc-lmb.cam.ac.uk/personal/pemsley/coot/) (58). The stereo chemical quality of the model was confirmed using a Ramachandran plot. Molecular diagrams were drawn using the PyMOL molecular graphics suite3 (https://pymol.org/2/). The final model contains residues 716 to 1032 of Cdk12, residues 20 to 260 of CycK with one molecule SR-4835 bound to Cdk12. The model has been refined to R_work_ and R_free_ values of 24.8% and 26.4%, respectively. Details of the diffraction data collection, quality, and refinement statistics are given in Table S1. The atomic coordinates and structure factor amplitudes have been deposited in the Protein Data Bank with accession code 8P81.

### *In vitro* kinase assay

Radioactive kinase activity assays were performed in a total volume of 15 μl per sample in kinase assay buffer (50 mM Hepes/pH 7.6, 34 mM KCl, 7 mM MgCl_2_, 5 mM β-glycerol phosphate, and 2.5 mM dithioerythritol). Preincubation of 0.2 μM or 0.02 μM recombinant protein kinase with indicated concentrations of respective inhibitor and 0.2 to 2 mM ATP containing 0.45 mCi [^32^P]-γ-ATP/ml (PerkinElmer) was carried out for 5 min. Kinase reactions were started by addition of substrate protein (10 μM GST-Rb1 for Cdk4/CycD3, 10 μM GST-CTD_[52]_ for Cdk7/CycH/MAT1, 50 μM His-c-Myc for all others) and incubated for 20 min at 30 °C in a shaking incubator at 300 rpm. For pS7-CTD_[3]_ peptide, the substrate concentration was increased to 100 μM and assays were performed for 60 min. Reactions were terminated by the addition of EDTA to a final concentration of 50 mM. Reaction samples were separately spotted onto Amersham Protran nitrocellulose membranes (GE HealthCare) and subsequently washed three times for 5 min in PBS. Samples were transferred to 4 ml liquid scintillation vials and 2 ml liquid scintillator (Ultima Gold) were added. Radioactive counts were determined in a Beckman Liquid Scintillation Counter (Beckman Coulter) for 1 min. Measurements were conducted in triplicates and displayed as mean with SD. For analysis of dose response measurements and IC_50_ value determination, data of inhibitor-supplemented samples were normalized to DMSO solvent controls and their activity plotted against the inhibitor concentration. IC_50_ values were extracted from triplicate measurements by sigmoidal curve fitting using DataGraph (v. 5.0; https://www.visualdatatools.com/DataGraph/) or were determined by applying the Morrison quadratic equation for tight binding inhibitors using GraphPad Prism 10 (https://www.graphpad.com/) (40).

To determine Michaelis–Menten constants (*K*_*M*_) of ATP toward Cdk12/Cdk13 kinases, required for applying the Morrison quadratic equation to dose-response measurements, kinase assays were performed as described at constant substrate (50 μM His-c-Myc) and kinase (0.2 μM Cdk12/Cdk13) concentrations and varied ATP amounts (8.2, 25, 74, 222, and 666 μM ATP). Initial enzyme activity was determined by terminating reactions after 5 min by addition of EDTA. *K*_*M*_ values were then identified by plotting respective enzyme activity against ATP concentrations and utilizing the Michaelis–Menten kinetic fit incorporated in the GraphPad Prism 10 software.

### SPR measurements

SPR experiments were conducted on a Biacore 8K (Cytiva) device. The system was equilibrated in running buffer (20 mM Tris pH 8, 150 mM NaCl, 1 mM tris(2-carboxyethyl)phosphine, 0.1 mM MgCl_2_, 0.05% Tween20, and 2% DMSO) at 25 °C. Flow cell 1 and 2 surfaces of a CM5 sensor chip (Cytiva) were conditioned with 50 mM NaOH at a flow rate of 30 μl/min for 15 s and subsequently activated with a 1:1 mix of 0.1 M N-hydroxysuccinimide (NHS) and 0.1 M 3-(N,N-dimethylamino) propyl-N-ethyl-carbodiimide (EDC) at a flow rate of 10 μl/min for 7 min. The flow system was washed with 1 M enthanolamine (pH 8). WT and mutant kinases Cdk12/CycK, Cdk13/CycK and Cdk9/CycT1 were diluted in Mes (pH 6.5) to a final concentration of 2 μM. Immobilization of kinases was performed in running buffer without DMSO using the amine coupling method on flow cell 2 surface for 160 s at a flow rate of 10 μl/min. Free binding sites were blocked with 1 M ethanolamine pH 8.0 (10 μl/min) for 7 min. Kinetic binding experiments were performed in the single-cycle mode with injections of increasing concentrations of SR-4835 (1.25, 5, 20, 80, 320, 1280, and 5120 nM) or NVP-2 (0.078, 0.3125, 1.25, 5, 20, and 80 nM) with an association period of 120 s and a dissociation time of 300 s (20 μl/min). Data were collected at a rate of 10 Hz and were solvent corrected to compensate for any potential DMSO effects. For kinetic binding measurements, processed data were fitted using a 1:1 interaction model using the Biacore Insight Evaluation Software (Cytiva; https://www.cytivalifesciences.com/en/us/shop/protein-analysis/spr-label-free-analysis/spr-software-and-extensions/biacore-insight-evaluation-software-p-23528).

### Thermal protein stability analysis

For identification of thermal protein stability, nanoDSF experiments were conducted using a Prometheus NT.48 (NanoTemper) instrument. Proteins were diluted to 5 μM concentration in gel filtration buffer (see above) and supplemented with 2% DMSO, 1, 10, 100 μM SR-4835 for 10 min before start of the measurements. Changes in absorbance at the wavelengths λ = 330 and 350 nm were monitored at an excitation power of 25% in a temperature range from 20 to 90 °C with a slope of 1.5 °C/min. The measurement was conducted and evaluated with the PR.ThermControl software (https://nanotempertech.com/prometheus/nt48-software/). Experiments were conducted in duplicates.

### Western blots

For SDS-PAGE analysis, the recombinant protein samples (500 ng per lane) were mixed with SDS sample buffer and loaded onto a 12% SDS-PAGE gel. To transfer the proteins to an Amersham Protran nitrocellulose membrane (GE HealthCare), an assembly of an unstained SDS gel, nitrocellulose membrane, and filter paper was equilibrated in semi-dry blotting buffer (48 mM Tris, 39 mM glycine, 0.04% (v/v) SDS, and 20% (v/v) methanol) for 10 min. The assembly was then placed in a semidry blotting chamber V20 SDB (Scie-Plas). The transfer was conducted for 45 min at 0.16 A. Following the transfer, the nitrocellulose membrane was blocked for 1 h in 5% (w/v) milk in phosphate-buffered saline with Tween 20 (PBS-T). Next, a primary polyclonal antibody specific for Cdk12 phosphorylation site pT893 (AB-PK567, Kinexus) was applied at a 1:250 ratio in PBS-T and incubated at 4 °C overnight. As a secondary antibody, a mouse anti-rabbit immunoglobulin G antibody conjugated to horseradish peroxidase (Santa Cruz Biotechnology) was applied at a dilution of 1:5000 in PBS-T and incubated for 1 h at room temperature. To visualize the proteins, enhanced chemiluminescence solution was added to membranes for 1 min before imaging membranes using a charged-coupled device camera ChemiDoc XRS+ system (Bio-Rad).

## Data availability

All data in this study are available within the article, supporting information, and/or from the corresponding author on reasonable request.

## Supporting information

This article contains supporting information.

## Conflict of interest

All authors declare that they have no conflicts of interest with the contents of this article.
