# Supplementary material for: The reversible inhibitor SR-4835 binds Cdk12/cyclin K in a noncanonical G-loop conformation
Source: J Biol Chem. 2023 Nov 26;300(1):105501. doi: 10.1016/j.jbc.2023.105501 (PMC10767194; doi:10.1016/j.jbc.2023.105501)
Supplement: Supporting Figures S1–S6 and Tables S1 and S2 [file mmc1.pdf]

Supporting Information

The reversible inhibitor SR-4835 binds Cdk12/Cyclin K in a non-canonical G-loop conformation

Maximilian Schmitz[1], Ines H. Kaltheuner[1], Kanchan Anand[1], Robert Düster[1], Jonas Moecking[1], Andrii Monastyrskyi[2], Derek R. Duckett[2], William R. Roush[3], and Matthias Geyer[1,*]

From the [1]Institute of Structural Biology, University of Bonn, Venusberg-Campus 1, 53127 Bonn, Germany; [2]Department of Drug Discovery, Moffitt Cancer Center, Tampa, Florida 33612, USA; [3]Department of Chemistry, The Scripps Research Institute, Jupiter, Florida 33458, USA.

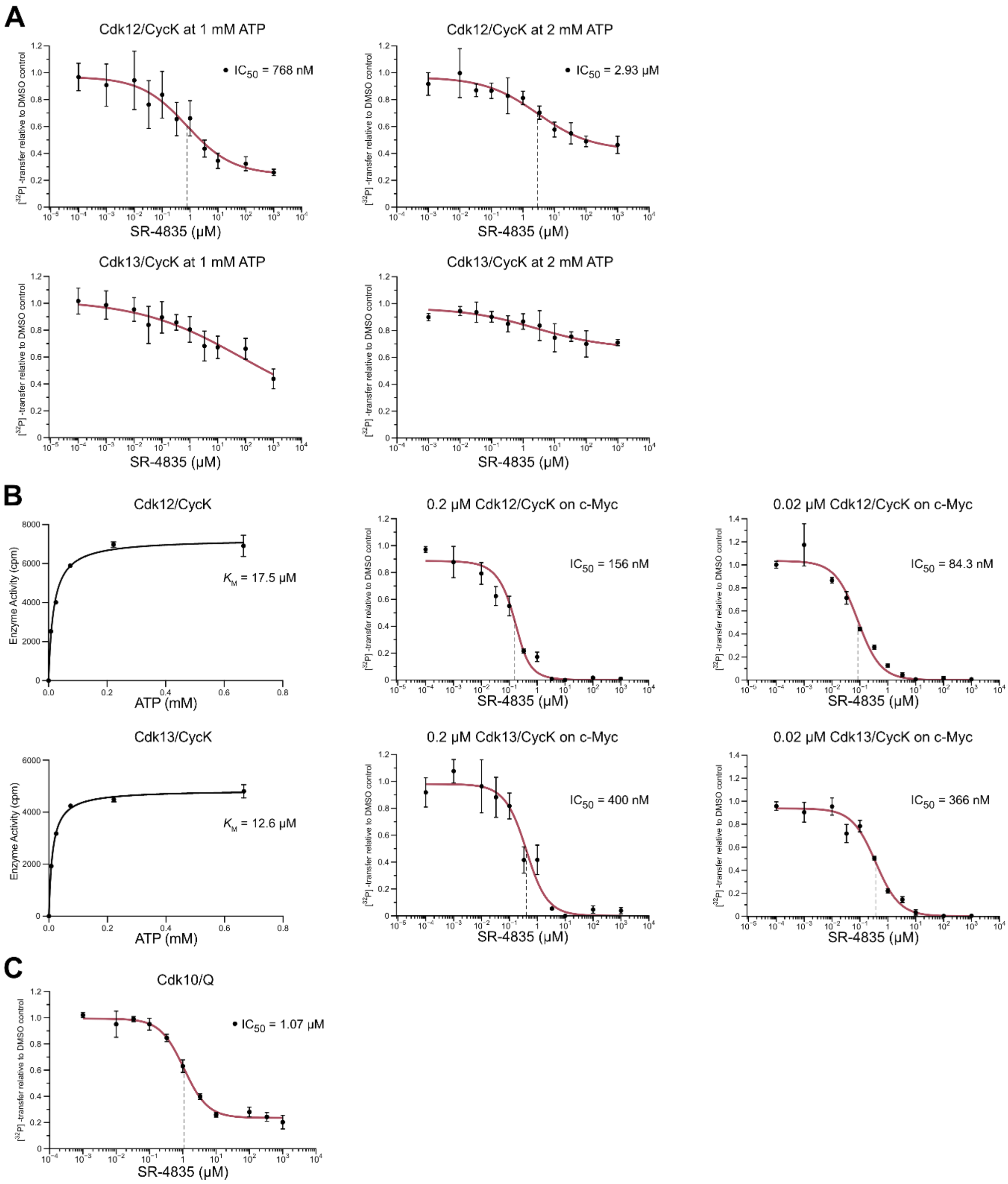

Figure S1. SR-4835's efficacy against Cdk12 and Cdk13 kinases applying different conditions. *A*, Dose-response measurements for the inhibition of Cdk12/CycK and Cdk13/CycK by SR-4835. Kinase assays were performed for a concentration series of SR-4835 in presence of 0.2 µM kinase and 1 or 2 mM ATP. Assays were started by addition of c-Myc and terminated after 20 min. Measurements were performed in triplicates (n = 3) and are depicted as mean ± SD. IC_{50} values were determined by sigmoidal curve fitting and are indicated by dashed lines. *B*, Evaluation of the dose-response measurements using the Morrison quadratic equation. K_M determination of ATP towards Cdk12 or Cdk13 was accomplished by performing kinase activity assays at constant substrate (50 µM c-Myc) and kinase (0.2 µM) concentrations, and varied ATP concentrations. Initial enzyme activities at 5 min were plotted against ATP concentrations and the Michaelis-Menten kinetic fit was applied to determine K_M values of 17.5 µM for Cdk12 and 12.6 µM for Cdk13, respectively. Dose-response measurements of SR-4835 with two indicated Cdk12/Cdk13 kinase concentrations on c-Myc substrate were analyzed, utilizing the quadratic equation by Morrison. Data is illustrated as mean ± SD from triplicates (n = 3) and calculated IC_{50} values are displayed as dashed lines. *C*, Kinase activity assays were conducted as in (*A*) at 0.2 mM ATP using a concentration series of SR-4835 for the inhibition of Cdk10/CycQ. Data is illustrated as mean ± SD from triplicates (n = 3).

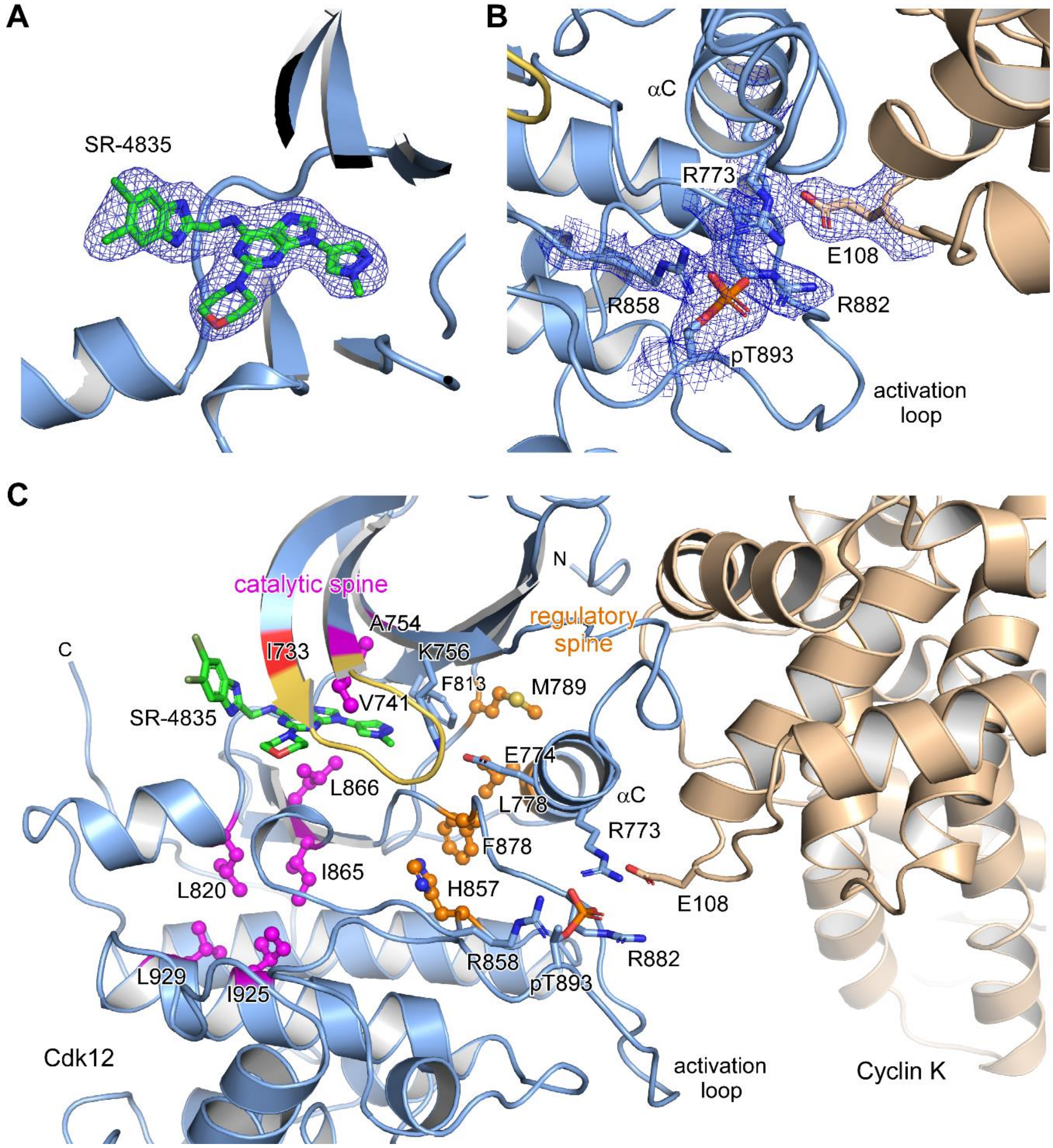

Figure S2. Structural features of the Cdk12•SR-4835/CycK complex.
A, Omit map for compound SR-4835 bound to Cdk12, contoured at 3.0 σ of the crystal structure at 2.68 Å resolution. *B*, Close up of the phospho-threonine pT893 coordination in the Cdk12 activation loop. The final $2F_o–F_c$ electron density is displayed at 1σ (left). *C*, Details of the Cdk12 kinase features when bound to SR-4835. The sides chains of the catalytic and regulatory spine residues are shown in ball-and-stick representation. The gatekeeper residue F813, which connects the two spines, and the catalytic lysine residue K756, which forms a salt bridge with E744 of the ^{768}PITAIRE motif, are highlighted. The coordination of the phospho-threonine pT893 is shown.

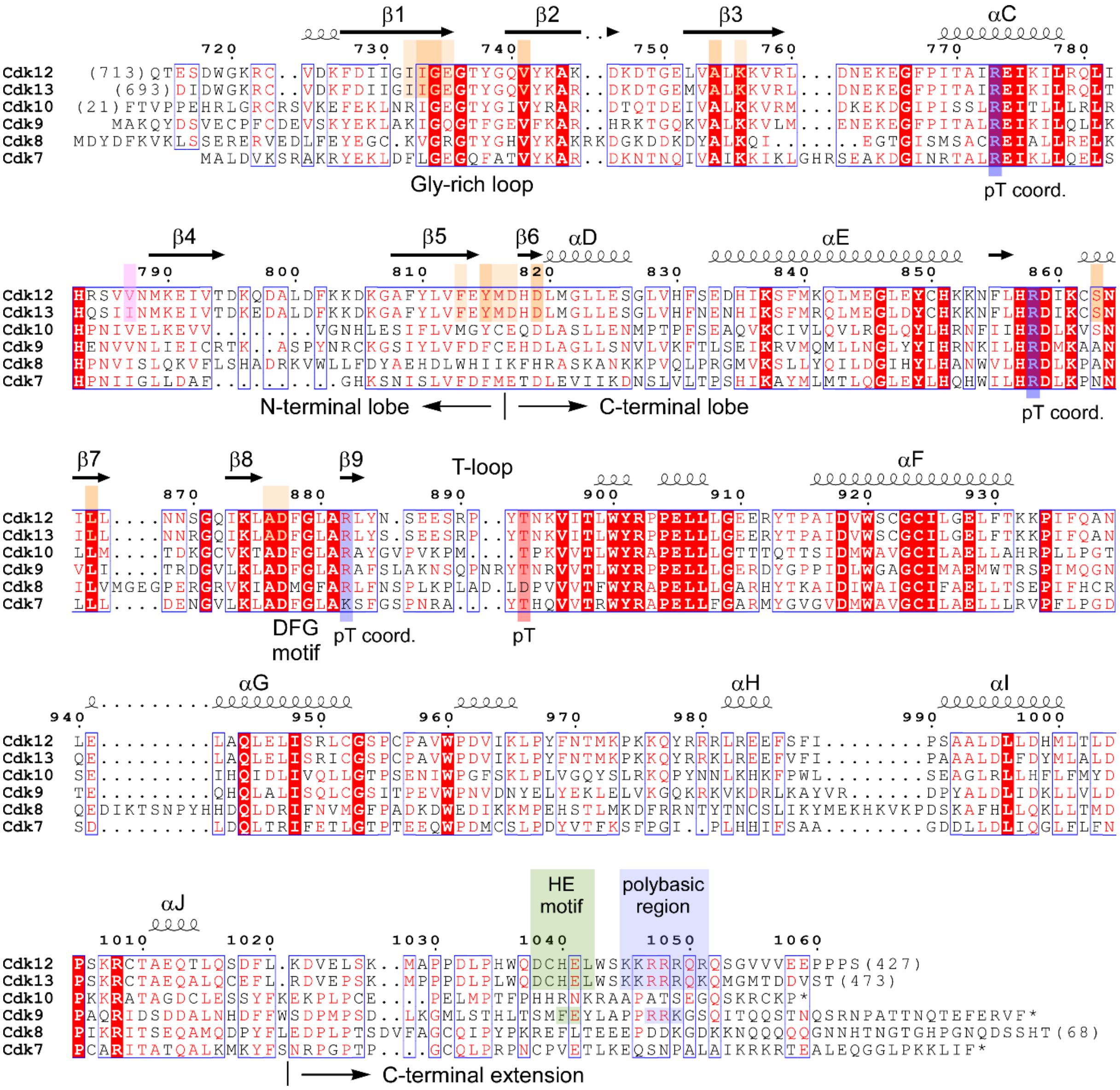

Figure S3. Sequence alignment of transcription-associated kinases Cdk7, -8, -9, -10, -12 and -13 and interacting residues of Cdk12 with SR-4835. Sequence alignment of the kinase domains of human CDKs 7-10, 12 and 13 based on the crystallized Cdk12 construct (714-1063). Secondary structure elements for Cdk12 and characteristic sequence motifs including the phosphorylated threonine within the T-loop (pT) and phosphorylated threonine-coordinating arginines (pT coord.) as well as functional regions are indicated. Residues which are boxed red are conserved in all kinases, similar residues are marked as red characters. The sequence alignment was performed with MultiAlin and secondary structure determination was done with ESPript. UniProt accession numbers are: Q9NYV4 (Cdk12), Q14004 (Cdk13), Q15131 (Cdk10), P50750 (Cdk9), P49336 (Cdk8), P50613 (Cdk7). Residues in Cdk12 mediating direct interactions with SR-4835 are boxed light and dark beige according to the buried surface area (5–12 and >12 Å^2, respectively) as determined with PDBePISA. The position of the one deviating residue in the binding interface to the compound, V787 in Cdk12 corresponding to I765 in Cdk13, is marked purple. Of note, V787 contributes only 2.5 Å^2 to the buried surface area.

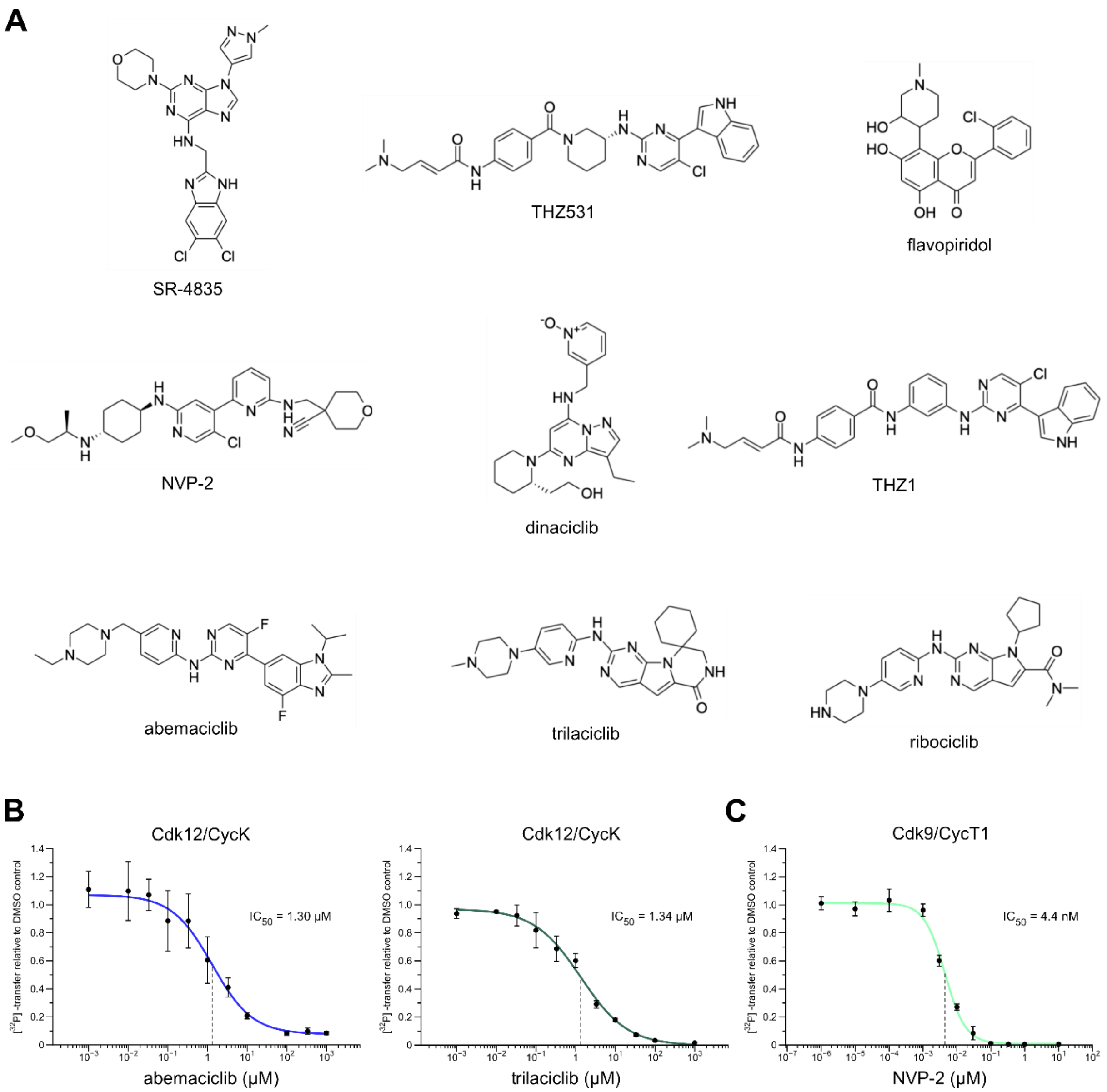

Figure S4. Chemical structures of the compounds tested against Cdk12/CycK. *A*, All depicted small molecule inhibitors were examined for their ability to reduce Cdk12/CycK activity in an *in vitro* kinase assay. Dinaciclib and NVP-2 displayed highest potency to inhibit Cdk12/CycK activity, while all other compounds (THZ1, SR-4835, THZ531, flavopiridol, abemaciclib, trilaciclib), with the exception of palbociclib and ribociclib, showed modest effects to reduce Cdk12/CycK activity. *B*, Dose-response measurements of the inhibitory effect of abemaciclib and trilaciclib against Cdk12/CycK. *C*, Dose-response measurements of the inhibitory effect of NVP-2 against Cdk9/CycT1. Radioactive kinase activity measurements in *B* and *C* were carried out as triplicates (n = 3) and are shown as mean ± SD. IC_{50} values were determined by sigmoidal fits and are indicated by dashed lines.

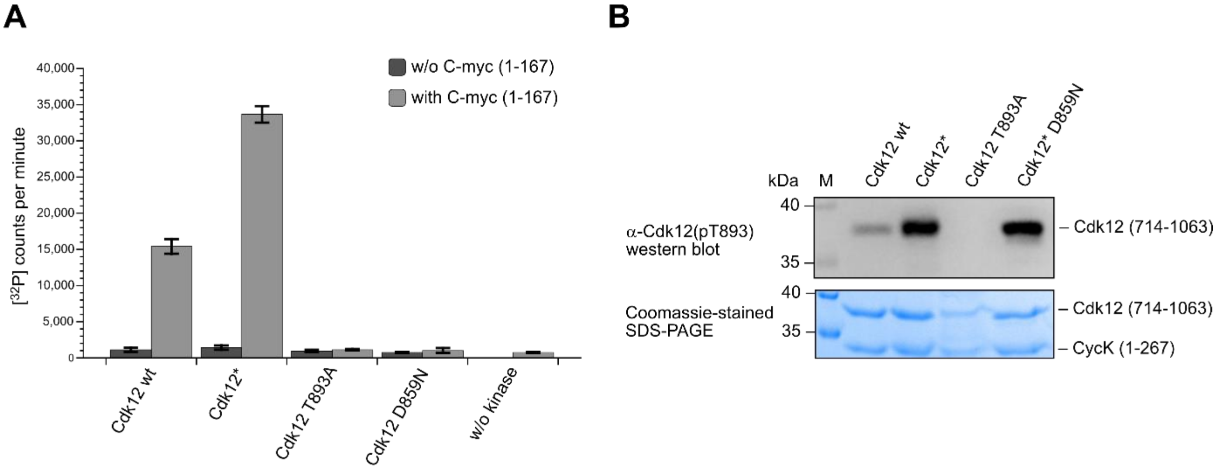

Figure S5. Kinase activity assays and pT893 western blot analysis of different wildtype and mutant Cdk12/CycK preparations. *A*, For radioactive kinase assays, 50 µM His_6-c-Myc substrate protein was incubated with 0.2 mM [^{32}P]-γ-ATP either with or without 0.2 µM kinase for 20 min. Samples with kinase without substrate protein functioned as additional negative controls. While wildtype Cdk12 (without CAK1 co-expression) displays ~15,000 cpm, an increase in activity is observed for CAK1 co-expressed Cdk12* (~35,000 cpm). T-loop mutant Cdk12 T893A and kinase dead mutant Cdk12 D859N show no activity in radioactive kinase assays. *B*, A Cdk12 pT893 specific antibody was used to visualize T-loop phosphorylation of different wildtype and mutant Cdk12 preparations.

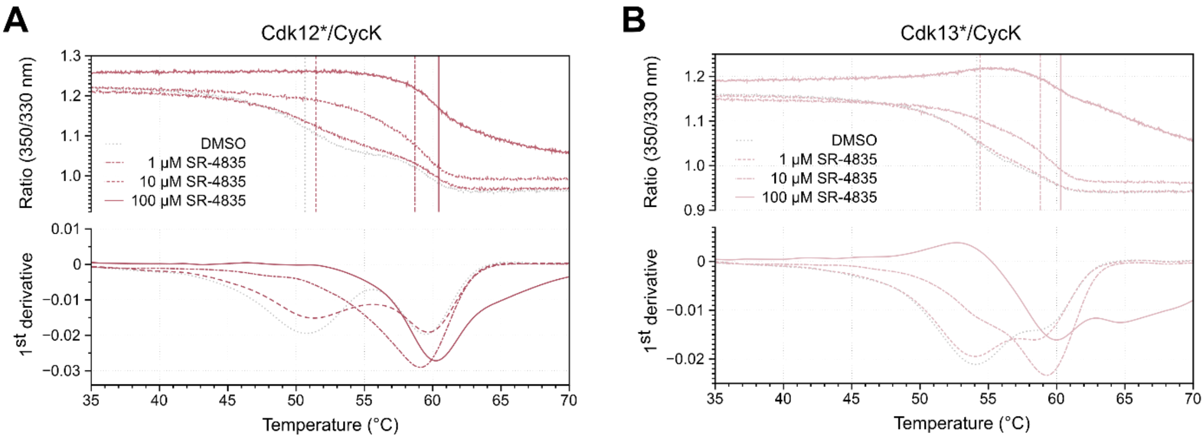

Figure S6. Raw data of nanoDSF measurements to determine thermal stability effect of SR-4835 towards Cdk12/CycK and Cdk13/CycK. NanoDSF measurements were performed to evaluate the thermal protein stability of *A*, Cdk12/CycK and *B*, Cdk13/CycK in absence and presence of SR-4835. 5 µM respective protein was supplemented with either 2 % DMSO or 1, 10, 100 µM SR-4835 as indicated and incubated for 10 min before measurements. Blots display changes in absorbance (ratio of 350/330 nm wavelength) and its 1st derivative in the range of 35-70°C. Lines correspond to inflection points of respective ratio, resulting in maximum/minimum in 1st derivative indicating melting temperatures of respective protein.

Table S1. Data collection and refinements statistics

	**Cdk12•SR-4835/CycK**
Data collection [a]	
Beam line	P14, DESY
Wavelength [Å]	0.9763
Space group	P 2_1 2_1 2
Unit cell: a, b, c [Å]<br>α, β, γ [°]	103.042, 137.906, 58.471<br>90, 90, 90
Resolution range [Å]	57.31 - 2.68 (2.77 - 2.68)
Unique reflections	21,122 (132)
Multiplicity	12.8 (10.1)
Completeness (%)	50.3 (6.0)
Mean I/sigma(I)	12.4 (0.1)
R_{meas}	0.0907 (2.027)
$CC_{1/2}$	1 (0.26)
Reflections used in refinement	12,093 (132)
Reflections used for R-free	1214 (14)
Refinement	
Model content	A: Cdk12 (716–1035), SR-4835<br>B: Cyclin K (22–260)
# of atoms macromolecules	4520
# of ligands	34
# of solvent	24
R_{work}	0.21 (0.50)
R_{free}	0.25 (0.49)
RMS deviations bonds [Å]	0.003
RMS deviations angles [°]	0..60
Ramachandran favored (%)	94.90
Ramachandran allowed (%)	4.2
Average B-factor	125.1
Macromolecules	125.2
ligands	117.55
solvent	114.9
PDB accession code	8P81

Table S2. Thermal stability measurements of Cdk12 and Cdk13 with SR-4835

Protein[a]	Treatment	T_m (°C)	ΔT_m to DMSO control (°C)
Cdk12/CycK (5 µM)	2% DMSO	50.65	
	1 µM SR-4835	51.45	+0.80
	10 µM SR-4835	58.70	+8.05
	100 µM SR-4835	60.45	+9.80
Cdk13/CycK (5 µM)	2% DMSO	54.15	
	1 µM SR-4835	54.40	+0.25
	10 µM SR-4835	58.80	+4.65
	100 µM SR-4835	60.30	+6.15

[a] Cdk12/CycK and Cdk13/CycK kinases were used at a final concentration of 5 µM. Control experiments as well as SR-4835 samples were measured at a concentration of 2% DMSO. Melting temperatures (T_m) were determined with the nanoDSF method on a Prometheus (NanoTemper) device and are depicted as means of two independent measurements.
